# Adherens Junction Formation Inhibits Lentivirus Entry and Gene Transfer

**DOI:** 10.1371/journal.pone.0079265

**Published:** 2013-11-13

**Authors:** Roshan Padmashali, Hui You, Nikhila Karnik, Pedro Lei, Stelios T. Andreadis

**Affiliations:** 1 Bioengineering Laboratory, Department of Chemical and Biological Engineering, University at Buffalo, State University of New York, Amherst, New York, United States of America; 2 Department of Biomedical Engineering, University at Buffalo, State University of New York, Amherst, New York, United States of America; 3 Center of Excellence in Bioinformatics and Life Sciences, Buffalo, New York, United States of America; Mayo Clinic, United States of America

## Abstract

Although cellular signaling pathways that affect lentivirus infection have been investigated, the role of cell-cell interactions in lentiviral gene delivery remains elusive. In the course of our studies we observed that lentiviral gene transfer was a strong function of the position of epithelial cells within colonies. While peripheral cells were transduced efficiently, cells in the center of colonies were resistant to gene transfer. In addition, gene delivery was enhanced significantly under culture conditions that disrupted adherens junctions (AJ) but decreased upon AJ formation. In agreement, gene knockdown and gain-of-function approaches showed that α-catenin, a key component of the AJ complex prevented lentivirus gene transfer. Using a doxycycline regulatable system we showed that expression of dominant negative E-cadherin enhanced gene transfer in a dose-dependent manner. In addition, dissolution of AJ by doxycycline increased entry of lentiviral particles into the cell cytoplasm in a dose-dependent manner. Taken together our results demonstrate that AJ formation renders cells non-permissive to lentiviral gene transfer and may facilitate development of simple means to enhance gene delivery or combat virus infection.

## Introduction

Recombinant lentivirus (LV) gained impetus in recent years as gene delivery vehicle due to its ability to transduce non-dividing cells and yield long-term gene expression with stable integration into the host genome. Pseudotyping with VSV-G (vesicular stomatitis virus glycoprotein) has further endowed broad tropism to LV enabling efficient gene transfer or gene knockdown strategies, development of transgenic models and more recently, generation of induced pluripotent stem cells [1]–[6]. VSV-G pseudotyped LVs are known to enter via clathrin mediated endocytosis while their interaction with target cells is attributed to charge based adsorption [7], [8]. Recently we implicated JNK signaling in LV entry where use of either chemical inhibitors or shRNA based JNK knockdown resulted in significant down-regulation of gene transfer efficiency [9]. Our previous work also showed that JNK played a crucial role in adherens junctions (AJ) formation by phosphorylating β-catenin and by controlling α-catenin binding to the AJ complex [10], [11]. Our findings implicating JNK in controlling AJ formation and in mediating LV entry prompted us to hypothesize that AJ formation/dissolution may affect LV transduction.

Epidermal AJ complexes consist of several proteins including α, β, γ, p120 catenins and transmembrane protein E-cadherin. AJ are formed by the intercellular homotypic interactions of junctional complexes via calcium binding extracellular, EC1 domain of E-cadherin. They play an important role in a number of cellular processes including proliferation, polarity, differentiation, inflammation, and cancer cell metastasis [12]–[19]. They have also been shown to interact with foreign pathogens such as bacteria, fungi and viruses, usually hindering their entry into cells and tissues [20]–[24]. In this context, entry of infectious viruses such as herpes simplex viruses (HSV) and pseudorabies virus (PRV) has been shown to increase under conditions that disrupt AJ and liberate E-cadherin associated virus receptors such as Nectin-1 [25]. Tight junctions (TJs) and AJ are also known to partner with the entry receptors (i.e. CAR receptors) for coxsackie B virus and adenovirus [26]. Others reported that primary ovarian cancer cells were more susceptible to infection by oncolytic adenovirus while undergoing epithelial-to-mesenchymal transition, a process that is accompanied by AJ dissolution [27]. On other hand, some viruses have evolved to disrupt intercellular adhesion. For example, rhinovirus infection was shown to disrupt AJ/TJs and abrogate the barrier function of nasal epithelium by impairing gene expression of junctional proteins [28]. Kaposi Sarcoma associated herpes virus (KSHV) is also known to degrade VE-Cadherin thereby increasing permeability of endothelial monolayers [29]. At high multiplicity of infection (MOI) lentivirus was shown to disrupt TJs of polarized airway epithelium, consequently decreasing trans-epithelial resistance (TER) [30]. Whether AJ play a similar critical role in mediating entry of non-replicating, recombinant lentivirus into non-polarized epithelial cells such as epidermal cells is yet to be established.

To this end, we employed chemical and genetic approaches (shRNA and dominant negative) to determine whether AJ affect lentivirus gene transfer. We found that the likelihood of gene transfer depended on the position of epithelial cells in a colony, being high in the periphery and low at the center of each colony. Gene transfer decreased significantly under conditions that favored AJ formation, and increased under conditions that induced AJ disruption or in the absence of junctional proteins that are required for a functional AJ complex. Using a doxycycline regulatable dominant negative E-cadherin expression system we demonstrated that gene transfer depended on the extent of AJ formation between epithelial cells. We also showed that AJ formation hindered gene delivery by preventing LV entry into target cells. Our results shed light into the role of AJ in LV-cell interactions and may have wider implications in gene therapy or development of strategies to combat viral infection.

## Materials and Methods

### Cell Culture

Human primary keratinocytes (hKCs) were isolated from neonatal foreskins as described before [31]. They were cultured and passaged routinely in keratinocyte-serum free medium supplemented with EGF and bovine pituitary extract (GIBCO Epilife Medium with 60 µM calcium, Life Technologies Corporation, Grand Island, NY). A431, ME180 and HEK 293T cells were cultured and passaged routinely in DMEM supplemented with 10% fetal bovine serum.

### Lentiviral Vectors for Gene Silencing and Over-expression

For gene silencing of α-E-Catenin, the shRNA sequence targeting α-E-Catenin: 5′-GGACCTGCTTTCGGAGTACAT-3′, which has been employed in our previous study, [10] was subcloned into the second-generation lentiviral vector pLVTHM (Addgene plasmid 12247; Addgene, Cambridge, MA). The resulting vector was further modified to replace the EGFP with the puromycin phosphotransferase selection gene to generate pLVTHM-sh-α-cat-Puro.

For E-Cadherin dominant negative (ECAD_DN) over-expression, a truncated form of E-Cadherin lacking the extracellular domain was PCR amplified with primers containing an additional N-terminal plasma membrane targeting sequence as described previously [32]. The PCR product was substituted for the EGFP in the lentiviral vector pCS-CG (Addgene plasmid 12154) using the NheI and AgeI sites to generate the pCSC-ECAD_DN vector.

Forward primer:

5′-CCTGCGCTAGCATGGGCAGCAGCAAGAGCAAGCCCAAGGACCCCAGCCAGAGA TTTCTTCGGAGGAGAGCGGTGGTCAA-3′.

Reverse primer:

5′-ACTTAACCGGTCTAGTCGTCCTCGCCGCCTCCGTA-3′.

Additionally, an IRES-Puromycin resistance fragment was inserted downstream of the ECAD_DN to generate the pCSC-ECAD_DN-IRES-Puro vector that was used for selection in mammalian cells. ECAD_DN was also subcloned into the pTRIPZ vector (Clontech, Mountain View, CA) between the MluI and AgeI restriction sites for doxycycline (Dox)-controlled expression generating the pTripZ-ECAD_DN vector.

To visualize actin fibers, the EGFP in pCS-CG was first replaced with a RFP variant DsRED Express2 (DRE2) to generate pCSC-DRE2. Subsequently the F-actin targeting sequence “Lifeact” [33] was fused to the N-terminal of DRE2 to generate the pCSC-lifeact-DRE2 vector. For expression of α-catenin/DRE2 fusion protein, wild type α-catenin was cloned downstream of DRE2 in pCSC-DRE2 to generate the pCSC-α-catenin/DRE2 fusion vector.

### Lentivirus Production and Transduction

For production of second-generation lentivirus, 293T/17 cells (ATCC, Manassas,VA) were transiently co-transfected with three plasmids using the standard calcium phosphate precipitation method [34]. Briefly, the following plasmids were mixed: 30 µg self-inactivating lentiviral vector pCS-CZsG encoding for ZsGreen driven by the CMV promoter (ZsGreen replaced EGFP in pCS-CG; Addgene plasmid 12154), 10 µg of envelope glycoprotein VSV-G encoding vector pMD2.G (Addgene plasmid 12259) and 25 µg of virus packaging plasmid psPAX2 (Addgene plasmid 12260). Upon calcium phosphate precipitation, this plasmid mixture was added to cells that had reached 70–80% confluence in T-150 flasks. After 20 hr transfection, the medium was replaced with fresh medium containing 5 mM sodium butyrate and viral supernatant was harvested 24 hr later. Virus supernatant was filtered using a 0.45 µm filter (Millipore, Bedford, MA) to remove cells debris and then concentrated by ultracentrifugation (50,000×g at 4°C for 2 hr). Finally, the virus was resuspended in keratinocyte-serum free medium and stored at −80°C. The same procedure was followed to produce lentiviruses for gene silencing or gene overexpression using the corresponding vectors described above instead of pCS-CZsG: pLVTHM-sh-α-cat, pCSC-ECAD_DN-IRES-Puro, pTripZ-ECAD_DN, pCSC-α-catenin/DRE2 fusion and pCSC-lifeact-DRE2.

For transduction, cells were plated in 48-well plates (1×10^4^ cells/cm^2^). One or two days later, purified lentivirus with 8 µg/ml polybrene was added to the cells for the indicated times. The virus was removed, the cells were washed twice with PBS and fresh medium was added. Gene transfer efficiency was measured as the fraction of ZsGreen+ cells using flow cytometry (BD FACSCalibur, BD, Franklin Lakes, NJ) and non-transduced cells served as negative control.

### Cell Lines

hKC-DRE2-actin was constructed by transducing hKCs with the lentiviral vector pCSC-lifeact-DRE2. These cells expressed a fusion protein of DRE2 with a 17 amino acid peptide that interacted with F-actin and was originally designed to follow actin dynamic in live cells [33]. Alpha-E-catenin deficient hKCs (hKC-sh-α-cat) or A431 cells (A431-sh-α-cat) were generated by transduction with the lentiviral vector pLVTHM-sh-α-cat. Following transduction, cells were selected in culture medium with 1 µg/ml puromycin for 6 days. Thereafter, cells were maintained in their corresponding medium without puromycin. Control, non-transduced cells did not survive beyond 3 days of exposure to puromycin. hKCs or A431 cells expressing dominant negative E-cadherin (hKC-ECAD_DN or A431-ECAD_DN) were generated by transduction with the lentiviral vector pCSC-ECAD_DN-IRES-Puro. Afterwards, cells were selected in culture medium with 1 µg/ml puromycin for 6 days. Similarly doxycycline (Dox)-regulatable ECAD_DN expressing hKCs or A431 cells were generated using the lentiviral vector pTripZ-ECAD_DN. Finally, ME180 overexpressing alpha-catenin/DRE2 fusion protein (ME180 α-cat) was generated as described in our earlier work [9]. Briefly ME180 was transduced with a lentivirus encoding for α-catenin/DRE2 fusion protein under the CMV promoter. The top 15% DRE2 expressing cells were sorted by flow cytometry and expanded before use in experiments.

### Immunofluorescence

Immunofluorescence was performed as described previously [11]. Briefly, cells were seeded on micro cover glass or 8-chamber glass slides in their respective media or experimental conditions. The cells were fixed with 4% paraformaldehyde for 10 min at 4°C, blocked and permeabilized with PBS containing 5% goat serum and 0.3% TritonX-100 for 1 hr at room temperature. The samples were incubated at 4°C overnight with primary antibodies in PBS, followed by secondary antibodies, Phalloidin and Hoechst the next day. The following antibodies and labeling agents were used: rabbit monoclonal anti-E-Cadherin (1∶200 in PBS; Clone 24E10, Cell Signaling #3195, Danvers, MA); mouse monoclonal anti-β-catenin (1∶200 in PBS; BD Biosciences #610153, San Jose, CA); secondary antibodies Alexa Fluor® 488/594 goat anti-rabbit/mouse (1∶400 in PBS with 5% goat serum; Life Technologies), Alexa Fluor® 488/594 Phalloidin for actin labeling (1∶50 in PBS; Life Technologies), and Hoechst 33342 for nuclear staining (Life Technologies). Cells were imaged with a Zeiss AxioObserver.Z1 (Carl Zeiss MicroImaging, Thornwood, NY) inverted fluorescence microscope equipped with an ORCA-ER CCD digital camera (Hammamatsu, Bridgewater, NJ).

### Western Blot Analysis of Internalized Viral Capsid Protein p24

Cells were washed once with cold PBS, trypsinized, centrifuged and washed with PBS to get rid of surface bound viral particles. The cell pellet was then lysed with lysis buffer (Cell Signaling) supplemented with dithiothreitol (DTT) and a cocktail of protease inhibitors (Roche Diagnostics, Mannheim, Germany). The lysates were then heated at 95°C for 5 min followed by centrifugation at 21,000×g for 5 min at 4°C. Proteins in the lysates were separated by SDS-PAGE at 200 V for 40 min (Mini-protean 3 system; BioRad Laboratories, Hercules, CA). Subsequently, protein samples were transferred to nitrocellulose membrane for 1 hr at 350 mA using an electrophoretic transfer cell (Mini Trans-Blot; Bio-Rad).

Membranes were blocked with blocking buffer containing 5% (wt/vol) nonfat dry milk in TBS-Tween (TBS-T: 20 mM Tris-HCl, pH 7.2–7.4, 150 mM NaCl, 0.1% vol/vol Tween 20) on a rocker platform for 1 hr at room temperature followed by overnight incubation with mouse anti-p24 (1∶1,000 in 5% nonfat dry milk; NIH AIDS Research and Reference Reagent Program, Germantown, MD) at 4°C. The following day, the membranes were incubated with horseradish peroxidase (HRP)-conjugated anti-mouse (1∶5,000 dilution in 5% nonfat dry milk, 1 hr at room temperature; Cell Signaling Technology) after being washed three times for removal of unbound primary antibody. The protein bands were detected using chemiluminescence (LumiGLO; KPL, Gaithersburg, MD) as per manufacturer’s instructions and exposed to KODAK film. Images were analyzed using Image J software (version 1.47, National Institute of Health, Bethesda, MD).

Western blotting for detecting α-catenin or dominant-negative E-Cadherin was performed similarly with cell lysates obtained from tissue culture plates. The following antibodies were used: rabbit monoclonal anti-E-Cadherin (1∶2,000 in TBS-T Buffer with 5%BSA; Clone 24E10, Cell Signaling#3195), mouse monoclonal anti-β-catenin (1∶2,000 in TBS-T Buffer with 5%BSA; BD Biosciences #610153, San Jose, CA).

### Statistical Analysis

Statistical analysis was performed using the statistical analysis tool in Microsoft Excel software. Values are means ± SE. Significant differences between treatment groups were determined by Student’s *t-test* (paired, two-tailed). Values of P≤0.05 were accepted as significant.

## Results

### Lentivirus Preferentially Transduced Cells in the Periphery of Epithelial Colonies

**Figure 1:**
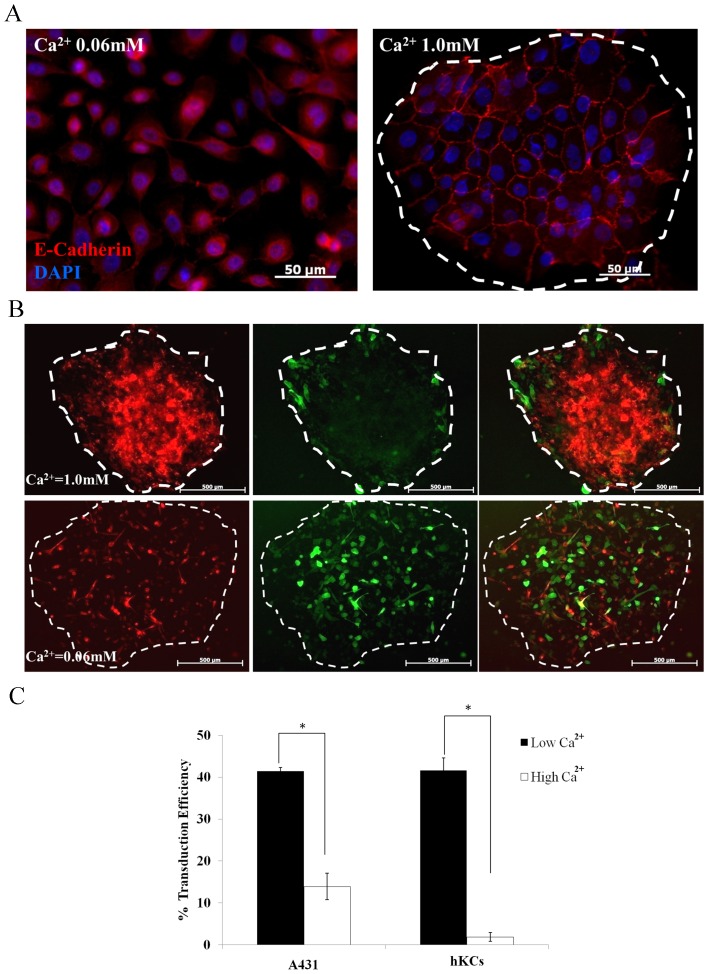
Calcium induces AJ formation and prevents LV transduction. (**A**) Human primary keratinocytes (hKCs) cultured on collagen-coated circular patterns in high (1 mM) and low (0.06 mM) Ca^2+^ containing medium were immunostained for E-cadherin (red) and the nuclei were counterstained with Hoechst (blue). Scale bar = 50 µm. (**B**) hKCs expressing DRE2-lifeact (hKC-DRE2-actin) were cultured on collagen-coated circular patterns in high (1 mM) and low (0.06 mM) Ca^2+^ containing medium and transduced with ZsGreen encoding lentivirus. Edges of circular patterns marked by dotted white lines. Scale bar = 500 µm. (**C**) hKCs and A431 cells were cultured in high (1 mM) and low (0.06 mM) Ca^2+^containing medium and transduced with ZsGreen encoding lentivirus. The percentage of transduced cells was measured by flow cytometry 3 days post transduction. All values represent the mean ± SD of triplicate samples in a representative experiment (n = 3). The asterisks (*) denote p<0.05 between low and high Ca^2+^ medium.

We examined whether formation of epithelial colonies through cell-cell adhesion affected lentivirus (LV) mediated gene delivery. Primary human keratinocytes (hKCs) were cultured on collagen pre-coated circular patterns in serum free medium containing high (1.0 mM) or low (0.06 mM) calcium (Ca^2+^) concentration, in order to promote or inhibit formation of Ca^2+^-dependent adherens junctions (AJ), respectively (shown by E-Cadherin localization in **Fig. 1A**). To facilitate visualization of the transduced cells in epithelial colonies using fluorescence microscopy, we employed hKCs expressing a fusion protein of DsRed Express2 (DRE2) with Lifeact (hKC-DRE2-actin), a 17 AA peptide that binds to F-actin and was shown to efficiently track actin dynamics without interfering with cytoskeletal function [33]. Colonies of hKC-DRE2-actin cells were transduced with ZsGreen encoding LV for 2 hr and two days later ZsGreen+ cells were visualized by fluorescence microscopy. Interestingly, under high Ca^2+^ medium cells were transduced preferentially in the periphery of each colony, while under low Ca^2+^ concentration, ZsGreen+ cells appeared uniformly distributed throughout the colonies (**Fig. 1B**).

### LV Transduction is Significantly Decreased at High Calcium Concentration

In order to quantify the effects of Ca^2+^ concentration on LV-mediated gene transfer, we transduced hKC and human vulva epidermoid carcinoma cells (A431), under low or high Ca^2+^ concentrations and the transduction efficiency (% ZsGreen+ cells) was measured by flow cytometry. For the carcinoma cell line A431, 42% cells were transduced in low Ca^2+^ medium as compared to only 14% under high Ca^2+^ concentration. The difference was even more pronounced for hKC, where the transduction efficiency decreased by about 20-fold from ∼42% at low to less than 2% under high Ca^2+^concentration **(** **Fig. 1C** **)**.

### ROCK Inhibition Compromised AJ and Increased LV Gene Transfer

**Figure 2:**
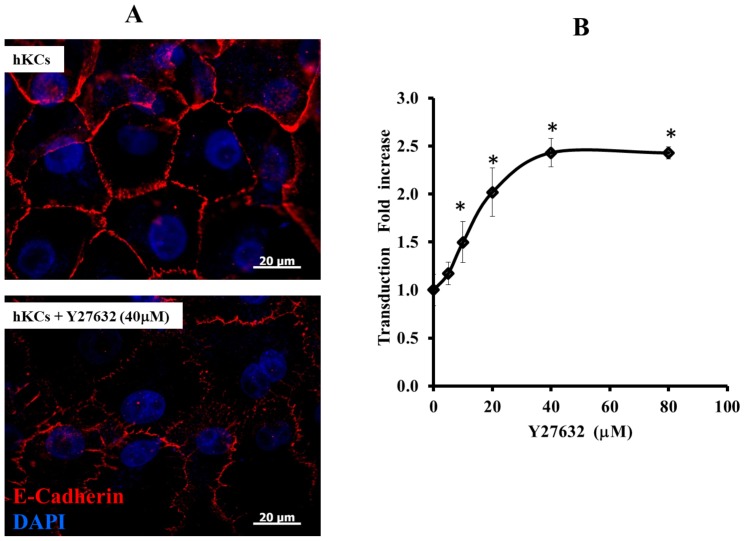
ROCK inhibition disrupts AJ and increases gene transfer. (A) hKCs cultured in high Ca^2+^ (1 mM) medium were treated with ROCK inhibitor Y27632 (40 µM) for 1 hr and stained for E-cadherin (red). The nuclei were counterstained with Hoechst (blue). Scale bar = 20 µm. (**B**) hKCs were cultured in high (1 mM) Ca^2+^ medium, treated with increasing concentrations of Y27632 (0–80 µM) for 30 min and transduced with ZsGreen encoding lentivirus for 2 hr. The percentage of transduced cells was measured by flow cytometry 3 days post transduction. All values represent the mean ± SD of triplicate samples in a representative experiment (n = 3). The asterisks (*) denote p<0.05 between the control (no Y27632) and the indicated Y27632 concentrations.

Since ROCK has been implicated in intercellular adhesion and the ROCK inhibitor, Y27632 was previously shown to disrupt AJ [35], we examined the effect of Y27632 on AJ formation and gene transfer. Indeed, E-Cadherin immunostaining showed that treatment with Y27632 (10 µM) had a dramatic effect on AJ, which appeared disrupted and punctated (**Fig. 2A**). This change in morphology was accompanied by a dose-dependent increase in transduction efficiency, which was ∼2.5 times higher at the highest Y27632 concentration (80 µM) (**Fig. 2B**). Collectively, these results suggested that formation of AJ had a significant effect on lentiviral gene transfer.

### AJ Formation Determines the Level of LV Gene Transfer

**Figure 3:**
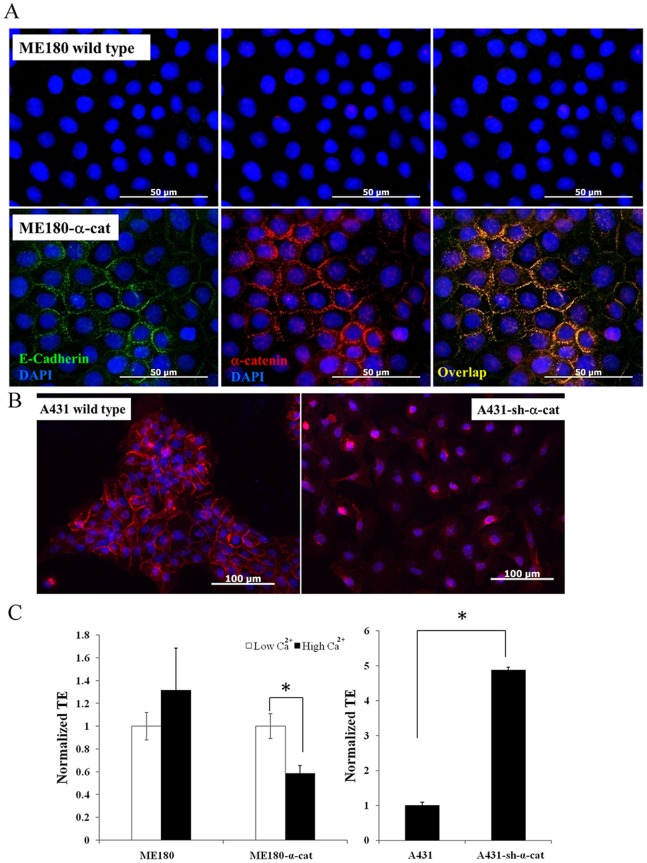
Effect of α-catenin knockdown and overexpression on LV transduction. (**A**) Wild type and α-catenin/DRE2 fusion protein over-expressing ME180 cells (ME180-α-cat) were cultured in high Ca^2+^ (1 mM) medium and stained for E-cadherin (green). The nuclei were counterstained with Hoechst (blue). The α-catenin/DRE2 fusion protein was visualized directly after fixation. Scale bar = 50 µm. (**B**) Wild type and A431-sh-α-cat were cultured in high Ca^2+^ medium, stained for E-cadherin (red) and the nuclei were counterstained with Hoechst (blue). Scale bar = 100 µm (**C**) Wild type ME180, ME180-α-cat, wild type A431 and A431-sh-α-cat cells were cultured in high Ca^2+^ (1 mM) or in low Ca^2+^ (0.06 mM) medium and transduced with ZsGreen-encoding lentivirus. The percentage of transduced cells was measured by flow cytometry 3 days post transduction. All values represent the mean ± SD of triplicate samples in a representative experiment (n = 3). The asterisks (*) denote p<0.05 between the indicated samples.

The results prompted us to *hypothesize that formation of AJ inhibits LV gene transfer*. To address this hypothesis we took a multi-prong approach that involves knockdown and gain-of-function approaches for key components of the AJ complex such as α-catenin and E-cadherin. It is well known that α-catenin was necessary for AJ formation in epidermal cells [10], [36]. Indeed, ME180 human cervical squamous cell carcinoma cells inherently lack the α-catenin gene and thereby they cannot form AJ, even under high Ca^2+^ conditions. However, upon introduction of the α-catenin/DRE2 fusion protein in these cells (ME180-α-cat) regained the capacity to form AJ as shown by co-localization of E-Cadherin with α-catenin/DRE2 at the cell-cell junction sites **(** **Fig. 3A** **)**. Interestingly, introduction of α-catenin/DRE2 fusion protein **(Fig. S1A)** decreased the gene transfer efficiency by more than 40% only under high Ca^2+^ conditions. In contrast, gene transfer to control ME180 cells lacking α-catenin was independent of Ca^2+^ concentration **(** **Fig. 3C** **)**.

In addition to gain-of-function, we also employed a knock down approach to examine the role of α-catenin in LV gene transfer. Specifically, knocking down α-catenin in A431 cells using shRNA **(Fig. S1B)** abolished AJ formation **(** **Fig. 3B** **)** and increased gene transfer by almost 5-fold **(** **Fig. 3C** **)** in comparison to wild type cells.

**Figure 4:**
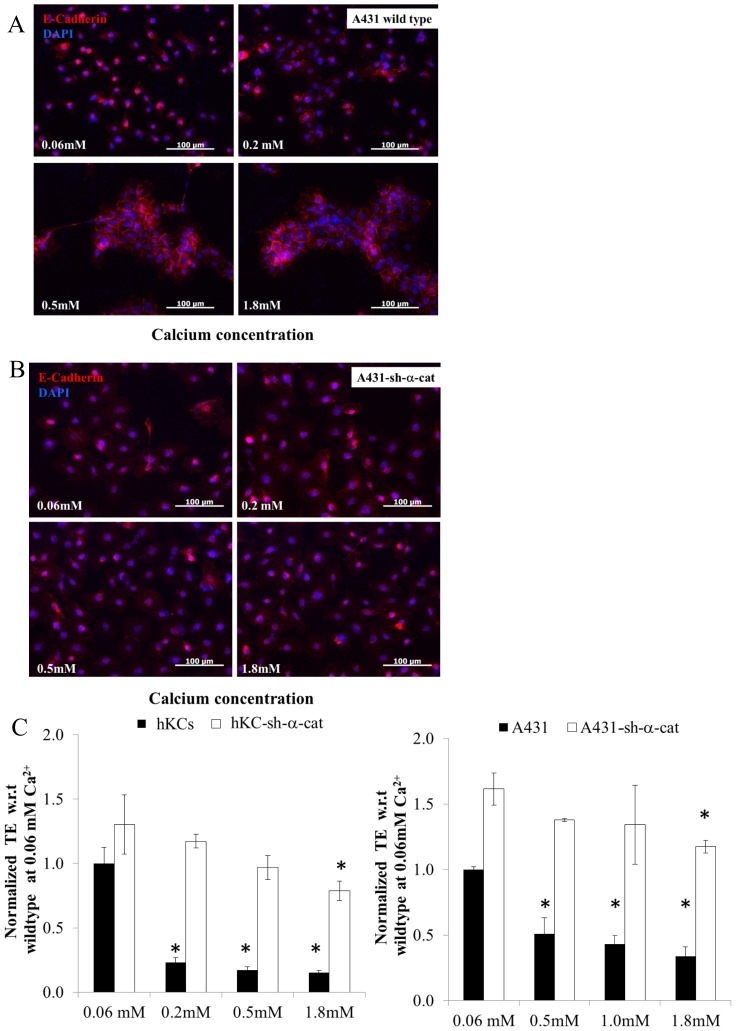
α-catenin knockdown disrupts AJ and increases LV transduction independent of Ca^2+^ concentration. (**A**) A431 cells cultured in serum free medium with increasing Ca^2+^ concentrations (0.06 mM-1.8 mM) were stained for E-cadherin (red) and the nuclei were counterstained with Hoechst (blue). (**B**) A431-sh-α-cat cells cultured in serum free medium with increasing Ca^2+^ concentrations (0.06 mM-1.8 mM) were stained for E-cadherin (red) and the nuclei were counterstained with Hoechst (blue). Scale bar = 100 µm. (**C**) Wild type and hKC-sh-α-cat or A431-sh-α-cat cells were cultured in serum free medium with increasing Ca^2+^concentrations (0.06 mM-1.8 mM) and transduced with ZsGreen-encoding lentivirus for 2 hr. The percentage of transduced cells was measured by flow cytometry 3 days post transduction. All values represent the mean ± SD of triplicate samples in a representative experiment (n = 3). The asterisks (*) denote p<0.05 between low (0.06 mM) and the indicated Ca^2+^ concentrations.

Interestingly, loss of α-catenin rendered AJ dissolution and LV transduction independent of Ca^2+^ concentration. Specifically, at low Ca^2+^ concentration (0.06 mM) E-cadherin localized in the cytoplasm of control hKCs and A431 cells; at 0.2 mM Ca^2+^ E-cadherin junctions appeared weak and discontinuous; while at Ca^2+^ concentration of 0.5 mM or higher E-cadherin appeared dense and continuous along the cell-cell contact sites **(** **Fig. 4A** **)**. Accordingly, the transduction efficiency decreased significantly as a function of Ca^2+^ concentration for both hKCs and A431 cells **(** **Fig. 4C** **)**.

On the other hand, knocking down α-catenin in hKCs (hKC-sh-α-cat) or A431 cells (A431-sh-α-cat) resulted in loss of AJ even at high Ca^2+^ concentration (1.8 mM) **(** **Fig. 4B** **)**. Accordingly, LV gene transfer remained high and independent of Ca^2+^ concentration for both hKC-sh-α-cat and A431-sh-α-cat cells **(** **Fig. 4C** **)**. Taken together, the gain-of-function and gene knock down approaches suggested that LV gene transfer to epithelial cells depended on AJ formation.

### Dominant Negative E-cadherin Reduced AJ and Increased LV Gene Transfer

**Figure 5:**
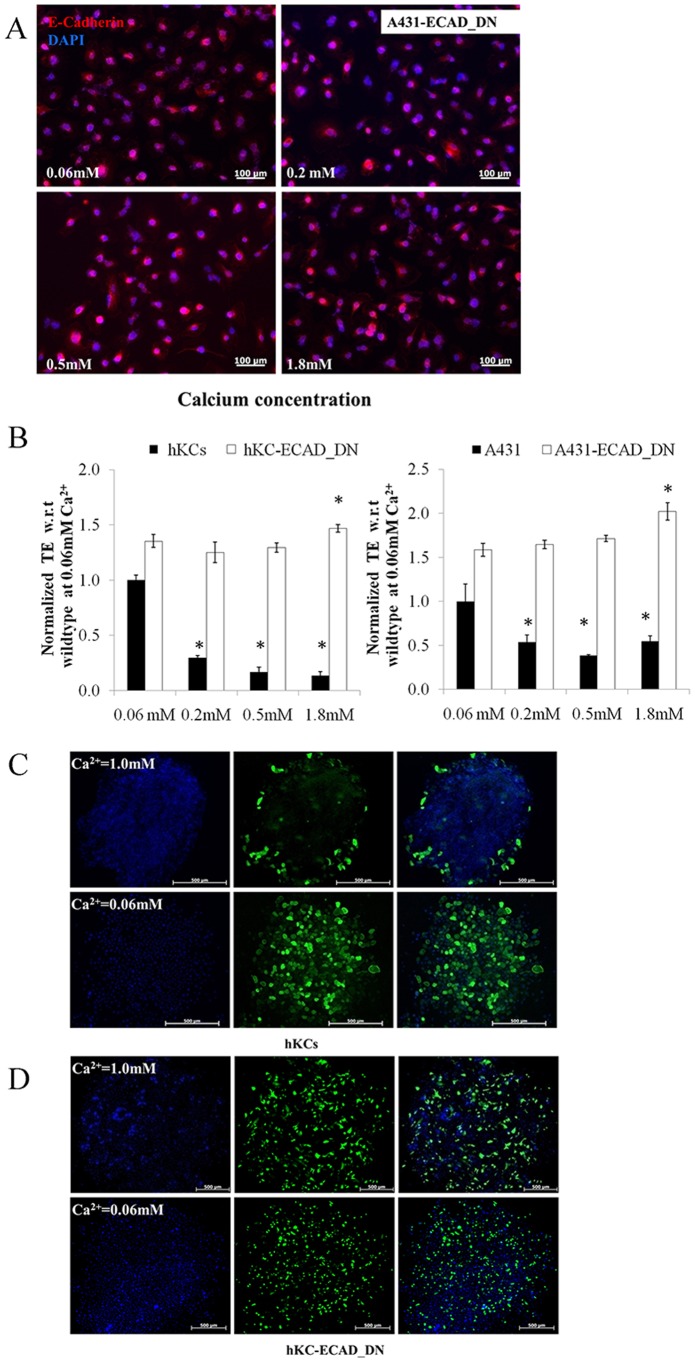
ECAD_DN overexpression disrupts AJ and increases LV transduction independent of Ca^2+^ concentration and position of cells within epithelial colonies. (**A**) A431-ECAD_DN cells were cultured in serum free medium with increasing Ca^2+^ concentrations (0.06 mM-1.8 mM) and stained for E-cadherin (red). The nuclei were counterstained with Hoechst (blue). (**B**) Wild type or hKC-ECAD_DN and A431-ECAD_DN cells were cultured in serum free medium with increasing Ca^2+^ concentrations (0.06 mM-1.8 mM) and transduced with ZsGreen encoding lentivirus for 2 hr. The percentage of transduced cells was measured by flow cytometry 3 days post transduction. All values represent the mean ± SD of triplicate samples in a representative experiment (n = 3). The asterisks (*) denote p<0.05 between the low (0.06 mM) and the indicated Ca^2+^ concentrations. (**C**) hKCs cultured on collagen-coated circular patterns in high (1 mM) or low (0.06 mM) Ca^2+^ containing medium and transduced with ZsGreen encoding lentivirus. Two days later the cell nuclei were counterstained with Hoechst (blue). Scale bar = 500 µm. (**D**) Same as in (C) with hKC-ECAD_DN. Scale bar = 500 µm.

To further explore the role of AJ in LV infection we overexpressed a dominant negative version of E-cadherin (ECAD_DN), which lacks the extracellular domain necessary for calcium mediated homotypic dimerization. Immunoprecipitation with anti β-catenin antibody showed that β-catenin predominantly bound to the dominant negative ECAD_DN (∼19 kDa) - instead of the full length E-cadherin (∼135 kDa) – possibly due to higher ECAD_DN concentration **(Fig. S1D)**. Similar to α-catenin knockdown, the ECAD_DN expressing hKCs or A431 cells were unable to form AJ at any Ca^2+^ concentration **(** **Fig. 5A** **)**. In agreement with the results using sh-α-cat cells, both ECAD_DN expressing hKCs and A431 cells exhibited significantly increased transduction efficiency, independent of Ca^2+^ concentration **(** **Fig. 5B** **)**. In addition, gene transfer to hKC colonies under high Ca^2+^ was limited to cells at the colony periphery. However, expression of ECAD_DN resulted in AJ-free colonies and transduced cells were uniformly distributed throughout the colony **(** **Fig. 5C, D** **)**, reminiscent of colonies grown under low Ca^2+^ concentration.

### LV Gene Transfer and Entry Depend on the Extent of Cell-cell Adhesion

**Figure 6:**
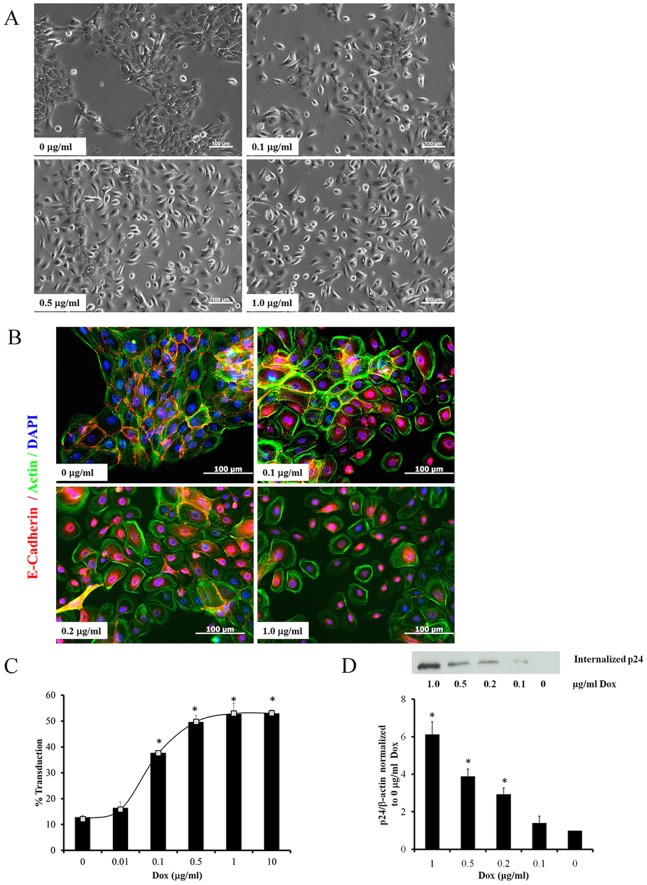
LV entry and transduction depend on the extent of AJ formation. (**A**) Phase contrast images of A431 cells expressing Dox-regulatable dominant negative E-cadherin (TripZ-ECAD_DN) in high Ca^2+^ (1 mM) containing medium at the indicated Dox concentrations. Scale bar = 100 µm. (**B**) TripZ-ECAD_DN expressing hKCs were cultured in high Ca^2+^ (1 mM) containing medium at the indicated Dox concentration and immunostained for E-cadherin (red), actin (green) and nuclei were counterstained with Hoechst (blue). (**C**) TripZ-ECAD_DN expressing hKCs were cultured in high Ca^2+^ (1 mM) containing medium at the indicated Dox concentration and transduced with ZsGreen encoding lentivirus for 2 hr. The percentage of transduced cells was measured by flow cytometry 3 days post transduction. All values represent the mean ± SD of triplicate samples in a representative experiment (n = 3). The asterisks (*) denote p<0.05 between the control (no Dox) and the indicated Dox concentration. (**D**) TripZ-ECAD_DN expressing hKCs were cultured in high Ca^2+^ (1 mM) containing medium at the indicated Dox concentrations and transduced with lentivirus for 30min. Then the cells were trypsinized to remove cell-surface bound virus and lysed. Internalized viral protein p24 was quantified by western blot and band intensity was measured by Image J, normalized to β-actin and plotted as a function of Dox concentration. All values represent the mean ± SD of triplicate samples in a representative experiment (n = 3). The asterisks (*) denote p<0.05 between the control (no Dox) and the indicated Dox concentration.

Based on these results *we hypothesized that LV gene transfer may depend on the extent of cell-cell adhesion*. To address this hypothesis, we expressed ECAD_DN through a doxycycline (Dox) regulatable expression vector (pTripZ-ECAD_DN) that was able to control the extent of ECAD_DN expression by varying the Dox concentration **(Fig. S1C)**. As control, cells were transduced with the empty vector, TripZ. After overnight treatment with Dox in the presence of high Ca^2+^, control cells treated with Dox (0 to 1 µg/ml) or TripZ-ECAD_DN expressing cells in the absence of Dox formed tight colonies. In contrast, at high Dox concentrations, ECAD_DN expressing cells failed to form colonies and remained as individual cells **(Fig. S2 and** **Fig. 6A** **)**. Accordingly, immunostaining for E-cadherin showed weakening of AJ with increasing Dox concentration **(** **Fig. 6B** **)**. In agreement, the transduction efficiency increased by more than 4-fold with increasing Dox concentration from 12% (no Dox) to 52% (1 or 10 µg/ml) **(** **Fig. 6C** **)**.

We also examined whether loss of AJ enhanced entry of LV particles in target cells. To this end, TripZ-ECAD_DN expressing A431 cells were treated with various Dox concentrations and the next day they were exposed to LV for a short time (30 min). After removal of the virus, the cells were trypsinized to remove surface-bound viral particles and pelleted cells were lyzed to measure internalized viral particles by WB for the capsid protein, p24. As shown in **Fig. 6D** very little – if any – p24 was detected in the absence of Dox but internalized p24 increased significantly with increasing Dox concentration, reaching 6-fold increase at 1 µg/ml of Dox. Collectively, our results show that formation of AJ prevented LV entry into epithelial cells and gene transfer; conversely loss of AJ by chemical or genetic means increased LV entry and gene transfer.

Finally, we examined whether the inhibition of LV gene transfer by AJ formation was dependent on the mode of viral entry. To this end, we engineered LV that was pseudotyped with the Moloney Murine Leukemia retrovirus amphotropic envelope, gp70 (ampho-LV) that is known to mediate virus entry via cell surface plasma membrane fusion. As shown in **Fig. S3**, ampho-LV gene transfer increased by 40% and 100% in A431-ECAD_DN and hKC-ECAD_DN, respectively, suggesting that lentivirus transduction, whether via receptor-dependent or -independent entry mode, is significantly affected by AJ formation in target cells.

## Discussion

In this study we attempted to understand whether intercellular adhesion through AJ affects LV-cell interactions and gene transfer. To this end, we employed a battery of approaches and showed that E-cadherin based AJ prevented efficient gene transfer by inhibiting virus entry into the cell cytoplasm. First, we transduced epithelial cells under conditions that favor (high Ca^2+^) or hinder (low Ca^2+^) formation of colonies through AJ-mediated cell-cell interactions. The peripheral cells of these colonies connected with their neighbors inside the colony but they had no AJ on their free edge facing outwards. Interestingly, we observed that LV-transduced cells were localized primarily around the periphery of these colonies, suggesting that viral entry sites may be more accessible at these peripheral cells as compared to cells in the colony center. Previous studies reported a similar bias for infection along the periphery of MDCKII cell colonies that were transduced with herpes simplex virus (HSV) [37], [38] as well as bacterial infections by *Listeria Monocytogenes* [39]. Conversely, we observed that when calcium was reduced to the level that did not support AJ, the bias was eliminated and transduced cells appeared to be distributed uniformly throughout the colony. In addition, the transduction efficiency increased significantly for A431 cells (4-fold) and was even higher for hKCs (10-fold), possibly because hKC formed stronger and continuous AJ with dense underlying cortical actin network as compared to relatively thinner and discontinuous AJ in the cancerous A431 cells. These results showed that Ca^2+^ concentration affected LV gene transfer through formation of AJ.

This hypothesis was further explored by inhibiting the ROCK pathway. Previous studies implicated RhoA and its downstream effector ROCK in controlling AJ formation/disassembly. The two isoforms, ROCK1 and ROCK2 seem to play distinct roles in controlling AJs; ROCK2 is required for junction disassembly via actomyosin contraction [40], while ROCK1 was recently reported to directly associate with the AJ complex [35]. Entry of other viruses, including the Ebola virus and vesicular stomatitis virus (but not HIV or adenoassociated virus 2) was significantly enhanced in cells with high expression of RhoC [41]. On the other hand, HSV-1 required active CDC42 to promote filopodia formation and provide the “rails” along which viral particles travel before successful entry [37], [42], [43]. In addition, ROCK1 depletion by shRNA or inhibition using Y27632 was shown to disrupt AJ and internalize junctional E-cadherin [35], [44]. Indeed, upon Y27632 treatment, hKCs and A431 cells assumed flattened morphology with poorly organized E-cadherin junctions even at high calcium concentration. What is more, weakening of junctions was accompanied by increased transduction in a Y27632 dose-dependent manner. These data supported a role of AJ in LV-cell interactions but direct evidence was still lacking.

To address this hypothesis directly, we disturbed intercellular adhesion by manipulating directly some of the components of the AJ complex. Among the AJ complex proteins, α-catenin plays a multifunctional role having binding domains for a number of junctional and cytoskeletal proteins such as β-catenin/plakoblobin, α-actinin, F-actin, vinculin as well as a homodimerization domain [45], [46]. In our experiments we employed gain-of-function as well as knockdown strategies to investigate the effect of α-catenin in AJ formation and gene transfer. First, we introduced α-catenin in the α-catenin deficient ME180 cells to enable formation of AJ, as shown by co-localization of α-catenin and E-cadherin at the cell-cell contact sites. AJ formation was accompanied by a decrease in gene transfer efficiency. Conversely, silencing of α-catenin in A431 cells resulted in disruption of AJ and significantly enhanced gene transfer, which was independent of the Ca^2+^ concentration, even up to 1.8 mM. This result suggested that manipulation of α-catenin had significant effect on LV gene transfer possibly by affecting AJ formation.

The direct effect of AJ formation/dissolution on gene transfer was further investigated by directly modulating the function of E-cadherin through overexpression of a dominant negative E-cadherin, which is a truncated form of E-cadherin lacking the ectodomain of the wild-type (wt) protein. It acts as dominant negative because it binds to E-cadherin cytoplasmic partners depleting them from the cytoplasm and decreasing their binding to wt E-cadherin [47], [48]. As a result it was expected to decrease the strength of cell-cell adhesion even in confluent monolayers. Indeed, hKCs expressing ECAD_DN failed to form AJ. As a result the efficiency of LV gene transfer increased significantly and transduced cells were not present only in the periphery of each colony but were distributed uniformly throughout. In addition, use of a Dox regulatable vector allowed us to express ECAD_DN in a dose-dependent manner leading to a dose-dependent increase in lentivirus entry into the cell cytoplasm and gene transfer. It will be interesting to employ Dox-regulatable, ECAD_DN expressing cells to generate 3D tissue models e.g. bioengineered epidermis with temporally controlled intercellular adhesion to study gene transfer in a 3D context.

Interestingly, this finding may have significant implications in lentiviral gene transfer to epidermal stem cells. The epidermis contains cells with different growth potential and at different stages of differentiation: slowly dividing stem cells that continue to proliferate for the lifetime of the tissue; transit amplifying (TA) cells that divide fast but are limited to a finite number of cell divisions before their progeny must commit to differentiate; and terminally differentiated cells, which will eventually reach full maturity and die. On fibroblast feeder layers, epidermal cells grow in colonies, containing all three types of cells, which are organized in concentric circles with stem cells in the inner core, TA cells surrounding stem cells and differentiated cells in the outer layers of each colony [49]–[51]. The stem cells are tightly packed and express high level of E-cadherin, which decreases in the TA cells and more so in the differentiated cells of the outer layers [50]. Our results showed that hKCs in the periphery of each colony i.e. differentiated cells were more likely to be transduced by ZsGreen-encoding LV and that culture in low calcium concentration prevented colony formation and increased gene transfer to more than 20 times. These results suggest that culture conditions that prevent cell-cell adhesion may increase lentiviral gene transfer to the epidermal stem compartment, and therefore, provide a simple means to increase the potential of genetically modified epithelial stem cells to treat short- or long-term disease states.

These results also suggested that intact epithelial tissues with strong intercellular adhesion might be more difficult to infect with LV and maybe other viruses as well. Indeed, it has been reported that junction integrity is weakened in the airway epithelium of asthmatic patients who exhibit increased susceptibility to respiratory viruses such as rhinovirus [52]. Disruption of bronchial epithelium with a function blocking E-Cadherin antibody (SHE78-7) or in the presence of low Ca^2+^ concentration resulted in significantly increased adenoviral infection [53]. Similarly, disruption of intercellular adhesion though chemical e.g. lysophosphatidylcholine (LPC) or mechanical means e.g. dynamic compression resulted in higher susceptibility of broncho-epithelial tissues to rhinovirus infection or lentivirus gene transfer [54]–[56]. In agreement, treatment of a cystic fibrosis mouse model with LPC was shown to increase LV gene transfer of the cystic fibrosis transmembrane conductance regulator (CFTR) gene and improve lung function [57], [58]. However, all these models are complicated by the use of chemicals or mechanical forces causing cellular damage that compounds data interpretation. Our work uses shRNA and dominant negative approaches targeting specific molecules in the AJ complex, thereby providing clear evidence that AJ directly affect LV entry and gene transfer.

The effect of AJ formation was not limited to vectors pseudotyped with the VSV-G envelope but extended to LV that was pseudotyped with the murine amphotropic retrovirus envelope, which enables virus entry via membrane fusion and not endocytosis. However, transduction by ampho-LV was seemingly affected to a lesser extent than transduction by VSV-G enveloped LV suggesting that LV entry by endocytosis may be more strongly dependent on cell-cell adhesion than entry by membrane fusion. Interestingly, during AJ formation the proteins participating in clathrin-mediated endocytosis (clathrin light chain, αAP2 subunit of AP2 complex) are recruited to the cell-cell junctions [59]. The depletion of endosome forming proteins from cell surface may be a reason for the reduction of LV endocytosis upon AJ formation. Alternatively, the highly organized network of actin bundles that are formed underneath the AJ may also provide a physical barrier to pathogen entry [60], [61], and possibly affect entry by endocytosis to a larger extent than entry by membrane fusion. Although the exact mechanism remains elusive, our results clearly establish the role of AJ in controlling the extent of LV infection and suggest that controlled disruption of intercellular junctions may provide a simple but efficient means of increasing gene transfer to epithelial cells and tissues.

## Supporting Information

Figure S1 **Immunoblotting showing overexpression or knockdown of AJ complex proteins. A)** ME180 cells deficient in α-catenin were transduced with lentivirus encoding for full-length α-catenin/DRE2 fusion. Western blot of lysates from wild type ME180 and ME180 α-cat cells; β-actin served as loading control. **B)** α-catenin was knocked down in A431 cells using shRNA encoding lentivirus. Western blot of lysates from wild type A431 and A431 sh-α-cat cells. **C)** Dox regulatable expression of ECAD_DN. Western blots of lysates from TripZ-ECAD_DN expressing hKCs treated with the indicated Dox concentrations. **D)** Immunoprecipitation of β-catenin in lysates from wild type hKCs or KC-ECAD_DN and WB for E-cadherin; β-catenin served as loading control. Note the difference in molecular weight of wild type and ECAD_DN.
(TIF)

Figure S2 **Doxycycline does not affect AJ.** Phase contrast images of hKCs transduced with control lentivirus (empty TripZ vector) in high Ca^2+^ (1 mM) medium at the indicated Dox concentrations. Scale bar = 100 µm.
(TIF)

Figure S3 **ECAD_DN overexpression increases transduction of LV pseudotyped with amphotropic retroviral envelope.** Wild type and ECAD_DN expressing A431 or hKCs were transduced with lentivirus pseudotyped with the amphotropic moloney murine leukemia virus envelope gp70 and encoded for GFP. The percentage of transduced cells was measured by flow cytometry 3 days post transduction. The asterisks (*) denote p<0.05 between and ECAD_DN expressing hKCs or A431 and their wild-type counterparts.
(TIF)

## References

[1] Naldini L (1999) In vivo gene delivery by lentiviral vectors. Thromb Haemost 82: 552–554. 10605750

[2] Kafri T (2004) Gene delivery by lentivirus vectors an overview. Methods Mol Biol 246: 367–390. 14970605 10.1385/1-59259-650-9:367

[3] Cockrell AS, Kafri T (2007) Gene delivery by lentivirus vectors. Mol Biotechnol 36: 184–204. 17873406 10.1007/s12033-007-0010-8

[4] Maherali N, Ahfeldt T, Rigamonti A, Utikal J, Cowan C, et al (2008) A high-efficiency system for the generation and study of human induced pluripotent stem cells. Cell Stem Cell 3: 340–345. 18786420 10.1016/j.stem.2008.08.003 PMC3987901

[5] Fu JD, Jung Y, Chan CW, Li RA (2008) An inducible transgene expression system for regulated phenotypic modification of human embryonic stem cells. Stem Cells Dev 17: 315–324. 18447646 10.1089/scd.2007.0114

[6] Brambrink T, Foreman R, Welstead GG, Lengner CJ, Wernig M, et al (2008) Sequential expression of pluripotency markers during direct reprogramming of mouse somatic cells. Cell Stem Cell 2: 151–159. 18371436 10.1016/j.stem.2008.01.004 PMC2276627

[7] Superti F, Seganti L, Ruggeri FM, Tinari A, Donelli G, et al (1987) Entry pathway of vesicular stomatitis virus into different host cells. J Gen Virol 68 (Pt 2): 387–399. 10.1099/0022-1317-68-2-387 3029282

[8] Johannsdottir HK, Mancini R, Kartenbeck J, Amato L, Helenius A (2009) Host cell factors and functions involved in vesicular stomatitis virus entry. J Virol 83: 440–453. 18971266 10.1128/JVI.01864-08 PMC2612308

[9] Lee MH, Padmashali R, Andreadis ST (2011) JNK1 is required for lentivirus entry and gene transfer. J Virol 85: 2657–2665. 21191018 10.1128/JVI.01765-10 PMC3067971

[10] Lee MH, Padmashali R, Koria P, Andreadis ST (2011) JNK regulates binding of alpha-catenin to adherens junctions and cell-cell adhesion. FASEB J 25: 613–623. 21030692 10.1096/fj.10-161380 PMC3023394

[11] Lee MH, Koria P, Qu J, Andreadis ST (2009) JNK phosphorylates beta-catenin and regulates adherens junctions. FASEB J 23: 3874–3883. 19667122 10.1096/fj.08-117804 PMC2774999

[12] Redfield A, Nieman MT, Knudsen KA (1997) Cadherins promote skeletal muscle differentiation in three-dimensional cultures. J Cell Biol 138: 1323–1331. 9298987 10.1083/jcb.138.6.1323 PMC2132549

[13] Theard D, Steiner M, Kalicharan D, Hoekstra D, van Ijzendoorn SC (2007) Cell polarity development and protein trafficking in hepatocytes lacking E-cadherin/beta-catenin-based adherens junctions. Mol Biol Cell 18: 2313–2321. 17429067 10.1091/mbc.E06-11-1040 PMC1877101

[14] Cheng SL, Lecanda F, Davidson MK, Warlow PM, Zhang SF, et al (1998) Human osteoblasts express a repertoire of cadherins, which are critical for BMP-2-induced osteogenic differentiation. J Bone Miner Res 13: 633–644. 9556063 10.1359/jbmr.1998.13.4.633

[15] Hodivala KJ, Watt FM (1994) Evidence that cadherins play a role in the downregulation of integrin expression that occurs during keratinocyte terminal differentiation. J Cell Biol 124: 589–600. 8106556 10.1083/jcb.124.4.589 PMC2119909

[16] James Nelson W (1996) Meeting of cell-cell adhesion, communication and signalling at the junction. Trends Cell Biol 6: 325–327. 15157442 10.1016/0962-8924(96)30040-8

[17] Knudsen KA, Frankowski C, Johnson KR, Wheelock MJ (1998) A role for cadherins in cellular signaling and differentiation. J Cell Biochem Suppl 30–31: 168–176. 9893268

[18] Orlichenko L, Weller SG, Cao H, Krueger EW, Awoniyi M, et al (2009) Caveolae mediate growth factor-induced disassembly of adherens junctions to support tumor cell dissociation. Mol Biol Cell 20: 4140–4152. 19641024 10.1091/mbc.E08-10-1043 PMC2754928

[19] Etienne-Manneville S (2012) Adherens junctions during cell migration. Subcell Biochem 60: 225–249. 22674074 10.1007/978-94-007-4186-7_10

[20] Sousa S, Lecuit M, Cossart P (2005) Microbial strategies to target, cross or disrupt epithelia. Curr Opin Cell Biol 17: 489–498. 16102958 10.1016/j.ceb.2005.08.013

[21] Bonazzi M, Cossart P (2011) Impenetrable barriers or entry portals? The role of cell-cell adhesion during infection. J Cell Biol 195: 349–358. 22042617 10.1083/jcb.201106011 PMC3206337

[22] Krishnan S, Fernandez GE, Sacks DB, Prasadarao NV (2012) IQGAP1 mediates the disruption of adherens junctions to promote Escherichia coli K1 invasion of brain endothelial cells. Cell Microbiol 14: 1415–1433. 22519731 10.1111/j.1462-5822.2012.01805.x PMC3410974

[23] Coyne CB, Bergelson JM (2006) Virus-induced Abl and Fyn kinase signals permit coxsackievirus entry through epithelial tight junctions. Cell 124: 119–131. 16413486 10.1016/j.cell.2005.10.035

[24] Bomsel M, Alfsen A (2003) Entry of viruses through the epithelial barrier: pathogenic trickery. Nat Rev Mol Cell Biol 4: 57–68. 12511869 10.1038/nrm1005 PMC7097689

[25] Yoon M, Spear PG (2002) Disruption of adherens junctions liberates nectin-1 to serve as receptor for herpes simplex virus and pseudorabies virus entry. J Virol 76: 7203–7208. 12072519 10.1128/JVI.76.14.7203-7208.2002 PMC136315

[26] Spear PG (2002) Viral interactions with receptors in cell junctions and effects on junctional stability. Dev Cell 3: 462–464. 12408795 10.1016/s1534-5807(02)00298-8

[27] Strauss R, Sova P, Liu Y, Li ZY, Tuve S, et al (2009) Epithelial phenotype confers resistance of ovarian cancer cells to oncolytic adenoviruses. Cancer Res 69: 5115–5125. 19491256 10.1158/0008-5472.CAN-09-0645 PMC2738419

[28] Yeo NK, Jang YJ (2010) Rhinovirus infection-induced alteration of tight junction and adherens junction components in human nasal epithelial cells. Laryngoscope 120: 346–352. 20013846 10.1002/lary.20764

[29] Qian LW, Greene W, Ye F, Gao SJ (2008) Kaposi’s sarcoma-associated herpesvirus disrupts adherens junctions and increases endothelial permeability by inducing degradation of VE-cadherin. J Virol 82: 11902–11912. 18815301 10.1128/JVI.01042-08 PMC2583667

[30] Castellani S, Di Gioia S, Trotta T, Maffione AB, Conese M (2010) Impact of lentiviral vector-mediated transduction on the tightness of a polarized model of airway epithelium and effect of cationic polymer polyethylenimine. J Biomed Biotechnol 2010: 103976. 20617131 10.1155/2010/103976 PMC2896616

[31] Bajaj BG, Lei P, Andreadis ST (2005) Efficient gene transfer to human epidermal keratinocytes on fibronectin: in vitro evidence for transduction of epidermal stem cells. Mol Ther 11: 969–979. 15922968 10.1016/j.ymthe.2004.10.023

[32] Nieman MT, Kim JB, Johnson KR, Wheelock MJ (1999) Mechanism of extracellular domain-deleted dominant negative cadherins. J Cell Sci 112 (Pt 10): 1621–1632. 10.1242/jcs.112.10.1621 10212155

[33] Riedl J, Crevenna AH, Kessenbrock K, Yu JH, Neukirchen D, et al (2008) Lifeact: a versatile marker to visualize F-actin. Nat Methods 5: 605–607. 18536722 10.1038/nmeth.1220 PMC2814344

[34] Tian J, Andreadis ST (2009) Independent and high-level dual-gene expression in adult stem-progenitor cells from a single lentiviral vector. Gene Ther 16: 874–884. 19440229 10.1038/gt.2009.46 PMC2714872

[35] Smith AL, Dohn MR, Brown MV, Reynolds AB (2012) Association of Rho-associated protein kinase 1 with E-cadherin complexes is mediated by p120-catenin. Mol Biol Cell 23: 99–110. 22031287 10.1091/mbc.E11-06-0497 PMC3248908

[36] Vasioukhin V, Bauer C, Degenstein L, Wise B, Fuchs E (2001) Hyperproliferation and defects in epithelial polarity upon conditional ablation of alpha-catenin in skin. Cell 104: 605–617. 11239416 10.1016/s0092-8674(01)00246-x

[37] Petermann P, Haase I, Knebel-Morsdorf D (2009) Impact of Rac1 and Cdc42 signaling during early herpes simplex virus type 1 infection of keratinocytes. J Virol 83: 9759–9772. 19640983 10.1128/JVI.00835-09 PMC2748048

[38] Schelhaas M, Jansen M, Haase I, Knebel-Morsdorf D (2003) Herpes simplex virus type 1 exhibits a tropism for basal entry in polarized epithelial cells. J Gen Virol 84: 2473–2484. 12917468 10.1099/vir.0.19226-0

[39] Temm-Grove CJ, Jockusch BM, Rohde M, Niebuhr K, Chakraborty T, et al (1994) Exploitation of microfilament proteins by Listeria monocytogenes: microvillus-like composition of the comet tails and vectorial spreading in polarized epithelial sheets. J Cell Sci 107 (Pt 10): 2951–2960. 10.1242/jcs.107.10.2951 7876360

[40] Samarin SN, Ivanov AI, Flatau G, Parkos CA, Nusrat A (2007) Rho/Rho-associated kinase-II signaling mediates disassembly of epithelial apical junctions. Mol Biol Cell 18: 3429–3439. 17596509 10.1091/mbc.E07-04-0315 PMC1951751

[41] Quinn K, Brindley MA, Weller ML, Kaludov N, Kondratowicz A, et al (2009) Rho GTPases modulate entry of Ebola virus and vesicular stomatitis virus pseudotyped vectors. J Virol 83: 10176–10186. 19625394 10.1128/JVI.00422-09 PMC2747995

[42] Clement C, Tiwari V, Scanlan PM, Valyi-Nagy T, Yue BY, et al (2006) A novel role for phagocytosis-like uptake in herpes simplex virus entry. J Cell Biol 174: 1009–1021. 17000878 10.1083/jcb.200509155 PMC2064392

[43] Van den Broeke C, Favoreel HW (2011) Actin’ up: herpesvirus interactions with Rho GTPase signaling. Viruses 3: 278–292. 21994732 10.3390/v3040278 PMC3185701

[44] Anderson SC, Stone C, Tkach L, SundarRaj N (2002) Rho and Rho-kinase (ROCK) signaling in adherens and gap junction assembly in corneal epithelium. Invest Ophthalmol Vis Sci 43: 978–986. 11923237

[45] Bajpai S, Correia J, Feng Y, Figueiredo J, Sun SX, et al (2008) {alpha}-Catenin mediates initial E-cadherin-dependent cell-cell recognition and subsequent bond strengthening. Proc Natl Acad Sci U S A 105: 18331–18336. 19017792 10.1073/pnas.0806783105 PMC2587611

[46] Capaldo CT, Macara IG (2007) Depletion of E-cadherin disrupts establishment but not maintenance of cell junctions in Madin-Darby canine kidney epithelial cells. Mol Biol Cell 18: 189–200. 17093058 10.1091/mbc.E06-05-0471 PMC1751327

[47] Dahl U, Sjodin A, Semb H (1996) Cadherins regulate aggregation of pancreatic beta-cells in vivo. Development 122: 2895–2902. 8787762 10.1242/dev.122.9.2895

[48] Onder TT, Gupta PB, Mani SA, Yang J, Lander ES, et al (2008) Loss of E-cadherin promotes metastasis via multiple downstream transcriptional pathways. Cancer Res 68: 3645–3654. 18483246 10.1158/0008-5472.CAN-07-2938

[49] Rheinwald JG, Green H (1975) Serial cultivation of strains of human epidermal keratinocytes: the formation of keratinizing colonies from single cells. Cell 6: 331–343. 1052771 10.1016/s0092-8674(75)80001-8

[50] Tudor D, Chaudry F, Harper L, Mackenzie IC (2007) The in vitro behaviour and patterns of colony formation of murine epithelial stem cells. Cell Prolif 40: 706–720. 17877611 10.1111/j.1365-2184.2007.00467.x PMC6496497

[51] Ruetze M, Gallinat S, Wenck H, Deppert W, Knott A (2010) In situ localization of epidermal stem cells using a novel multi epitope ligand cartography approach. Integr Biol (Camb) 2: 241–249. 20535415 10.1039/b926147h

[52] Holgate ST, Roberts G, Arshad HS, Howarth PH, Davies DE (2009) The role of the airway epithelium and its interaction with environmental factors in asthma pathogenesis. Proc Am Thorac Soc 6: 655–659. 20008870 10.1513/pats.200907-072DP

[53] Man Y, Hart VJ, Ring CJ, Sanjar S, West MR (2000) Loss of epithelial integrity resulting from E-cadherin dysfunction predisposes airway epithelial cells to adenoviral infection. Am J Respir Cell Mol Biol 23: 610–617. 11062139 10.1165/ajrcmb.23.5.4046

[54] Jakiela B, Brockman-Schneider R, Amineva S, Lee WM, Gern JE (2008) Basal cells of differentiated bronchial epithelium are more susceptible to rhinovirus infection. Am J Respir Cell Mol Biol 38: 517–523. 18063839 10.1165/rcmb.2007-0050OC PMC2358970

[55] Tomei AA, Choe MM, Swartz MA (2008) Effects of dynamic compression on lentiviral transduction in an in vitro airway wall model. Am J Physiol Lung Cell Mol Physiol 294: L79–86. 18024723 10.1152/ajplung.00062.2007

[56] Cmielewski P, Anson DS, Parsons DW (2010) Lysophosphatidylcholine as an adjuvant for lentiviral vector mediated gene transfer to airway epithelium: effect of acyl chain length. Respir Res 11: 84. 20569421 10.1186/1465-9921-11-84 PMC2905357

[57] Kremer KL, Dunning KR, Parsons DW, Anson DS (2007) Gene delivery to airway epithelial cells in vivo: a direct comparison of apical and basolateral transduction strategies using pseudotyped lentivirus vectors. J Gene Med 9: 362–368. 17380490 10.1002/jgm.1025

[58] Limberis M, Anson DS, Fuller M, Parsons DW (2002) Recovery of airway cystic fibrosis transmembrane conductance regulator function in mice with cystic fibrosis after single-dose lentivirus-mediated gene transfer. Hum Gene Ther 13: 1961–1970. 12427306 10.1089/10430340260355365

[59] Levayer R, Pelissier-Monier A, Lecuit T (2011) Spatial regulation of Dia and Myosin-II by RhoGEF2 controls initiation of E-cadherin endocytosis during epithelial morphogenesis. Nat Cell Biol 13: 529–540. 21516109 10.1038/ncb2224

[60] Delorme-Axford E, Coyne CB (2011) The actin cytoskeleton as a barrier to virus infection of polarized epithelial cells. Viruses 3: 2462–2477. 22355449 10.3390/v3122462 PMC3280511

[61] Vogelmann R, Amieva MR, Falkow S, Nelson WJ (2004) Breaking into the epithelial apical-junctional complex–news from pathogen hackers. Curr Opin Cell Biol 16: 86–93. 15037310 10.1016/j.ceb.2003.12.002 PMC3373727

