# Supplementary figures and images for: Adherens Junction Formation Inhibits Lentivirus Entry and Gene Transfer
Source: PLoS One. 2013 Nov 13;8(11):e79265. doi: 10.1371/journal.pone.0079265 (PMC3827380; doi:10.1371/journal.pone.0079265)

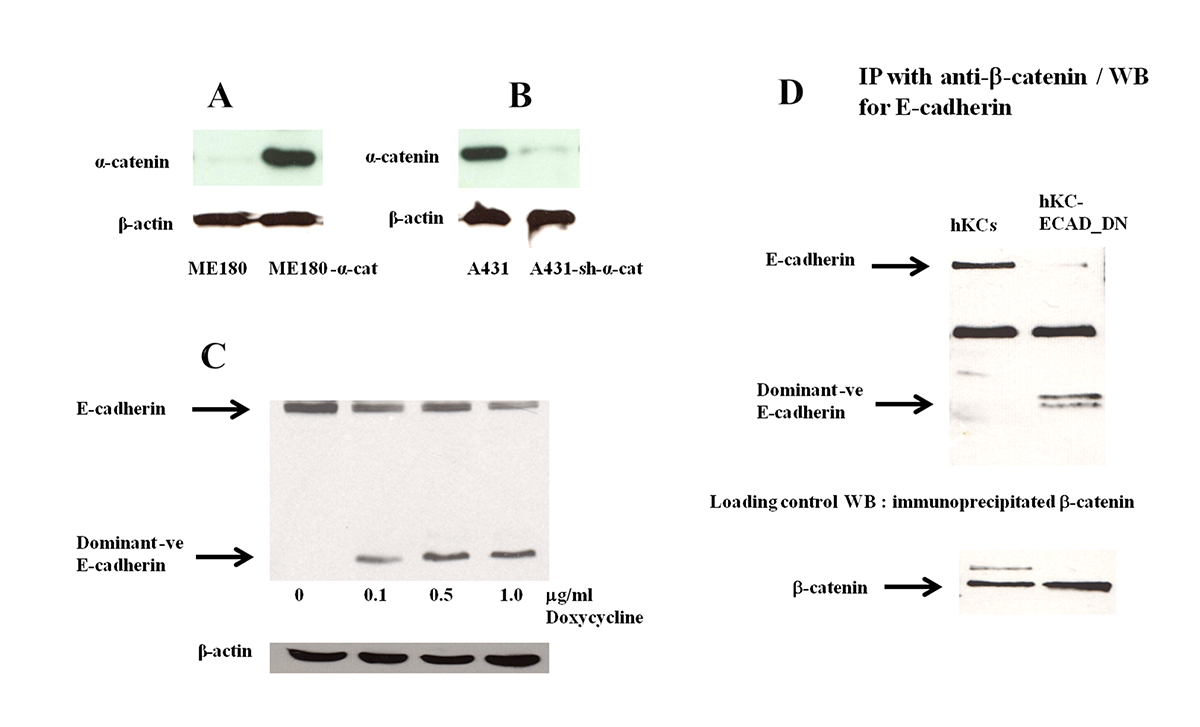

Supplement: Figure S1 — Immunoblotting showing overexpression or knockdown of AJ complex proteins. A) ME180 cells deficient in α-catenin were transduced with lentivirus encoding for full-length α-catenin/DRE2 fusion. Western blot of lysates from wild type ME180 and ME180 α-cat cells; β-actin served as loading control. B) α-catenin was knocked down in A431 cells using shRNA encoding lentivirus. Western blot of lysates from wild type A431 and A431 sh-α-cat cells. C) Dox regulatable expression of ECAD_DN. Western blots of lysates from TripZ-ECAD_DN expressing hKCs treated with the indicated Dox concentrations. D) Immunoprecipitation of β-catenin in lysates from wild type hKCs or KC-ECAD_DN and WB for E-cadherin; β-catenin served as loading control. Note the difference in molecular weight of wild type and ECAD_DN. (TIF) [file pone.0079265.s001.tif]
